# Proteomic Properties Reveal Phyloecological Clusters of *Archaea*

**DOI:** 10.1371/journal.pone.0048231

**Published:** 2012-10-25

**Authors:** Nela Nikolic, Zlatko Smole, Anita Krisko

**Affiliations:** 1 Mediterranean Institute for Life Sciences, Split, Croatia; 2 Institute of Biogeochemistry and Pollutant Dynamics, ETH Zurich, Zurich, Switzerland; 3 Department of Environmental Microbiology, Eawag, Duebendorf, Switzerland; 4 Institute of Cell Biology, ETH Zurich, Zurich, Switzerland; University of Cyprus, Cyprus

## Abstract

In this study, we propose a novel way to describe the variety of environmental adaptations of *Archaea*. We have clustered 57 *Archaea* by using a non-redundant set of proteomic features, and verified that the clusters correspond to environmental adaptations to the archaeal habitats. The first cluster consists dominantly of hyperthermophiles and hyperthermoacidophilic aerobes. The second cluster joins together halophilic and extremely halophilic *Archaea*, while the third cluster contains mesophilic (mostly methanogenic) *Archaea* together with thermoacidophiles. The non-redundant subset of proteomic features was found to consist of five features: the ratio of charged residues to uncharged, average protein size, normalized frequency of beta-sheet, normalized frequency of extended structure and number of hydrogen bond donors. We propose this clustering to be termed phyloecological clustering. This approach could give additional insights into relationships among archaeal species that may be hidden by sole phylogenetic analysis.

## Introduction

*Archaea* exhibit a large diversity of genotypic and phenotypic characteristics [1], [2]. Several different phenotypes have been described according to physicochemical properties of archaeal living habitats. Habitats are characterized by different concentrations of organic and inorganic substances and diverse composition of mini-atmosphere, all of which eventually shapes the metabolic profile of a species. Furthermore, some of archaeal organisms thrive in rather extreme conditions of temperature, salt concentration and/or pH. To mention only few environmental phenotypes: hyperthermophiles grow optimally at temperatures above 80°C [3]; thermophilic species have optimal growth between 45°C and 80°C; acidophiles have optimal pH ranging from 1.7 to 6.5 [4]; salt requirements of halophiles range from 1.5 M to extreme 5.2 M NaCl [5]; methanogens produce methane under anaerobic conditions [6], etc.

Archaeal species are thus highly adapted to life in various environments. Nucleic acids, proteins and other macromolecules inside a cell have to be, to a certain extent, adapted to particular conditions of the habitat where those unicellular organisms live. One could, therefore, assume that by looking at adaptation of macromolecules it would be possible to determine the conditions that an organism tolerates or prefers. Adaptation manifests at many different levels, and protein and whole proteome adaptation is surely one of them [7]–[14]. As the number of available annotated proteomes rapidly increases (currently over 1700 prokaryotic proteomes), it is a growing need to globally categorize species according to their lifestyles.

In this study we have focused exclusively on the adaptation of proteomes to diverse environmental conditions, by analyzing all proteins of archaeal species – both globular and membrane proteins, irrespective of protein families they belong to. We were interested in studying the relations between quantifiable characteristics of a proteomic sequence (such as average polarity, charge, hydrophobicity of the entire proteome, etc.) regarding conditions in the archaeal environmental niche. First, our goal was to define a non-redundant minimal set of proteomic descriptors that contain sufficient information about an adaptation to archaeal habitat. Second, we aimed to organize the diversity of archaeal environmental phenotypes and taxa based on the differences between their proteomes. For this purpose we used hierarchical clustering method, which has been widely applied in classification of protein sequences [15], [16], defining protein families [17], proteomic data mining [18], [19] and analyses of microarray data [20].

## Results and Discussion

### Non-redundant information about the proteomes

Each amino acid can be described with 544 different characteristics as listed in AAindex database ver.9.1 [21]. However, Atchley *et al*. [22] showed that these descriptors are highly redundant. Therefore, we first aimed to select a subset of features that would contain non-redundant information. In order to reduce the number of proteomic features to only non-redundant ones, we have developed a new feature selection procedure, which yielded the subset of original features that are uncorrelated. The procedure was unsupervised, which implies that we did not predetermine conditions or use any model to obtain the final subset. The final subset is the result of solely statistical approach that decreased the redundancy within the dataset. It should be emphasized at this point that we had not applied other commonly used methods (e.g. Principle Component Analysis) since we aimed at using the original proteomic features in the further analysis. Our intension was to retain full information on proteomic characteristics after feature selection procedure, in order to discuss more realistically about possible biological significance of the resulting proteomic feature subset.

Some previous studies [e.g. 23] have already generated proteomic feature subsets with the intent to classify organisms based on their environments. These were applications of supervised machine learning methods, where one only seeks answers for specific questions; for example, which proteomic features distinguish thermophiles from mesophiles. On the other hand, in our present study, we used unsupervised methods to generate the smallest possible subset of features that contains maximal information – the algorithm is not pre-trained to distinguish proteomic data according to environments. We sought not to pose any bias on the process of feature selection; our aim was to categorize adaptations to several (un)known conditions at once. *Archaea* have been found in many diverse environments, and to restrict the description of the environment on the currently known habitats could decrease the ability to categorize species yet to be discovered. For instance, the first discovered *Archaea* were extremophiles, while mesophilic *Archaea* were found to exist only in the last 20 years [24].

Our unsupervised feature selection generated the final subset containing five descriptors sufficient to discriminate *Archaea* according to their environmental adaptation. These are: average protein size, normalized frequency of beta-sheet (unweighted), normalized frequency of extended structure, the ratio of charged residues to uncharged, and number of hydrogen bond donors.

Based on the previously published data, thermophilic and hyperthermophilic proteins tend to be significantly shorter than mesophilic proteins [7], [25], [26]. Possible mechanisms could involve deletions in the exposed loop regions [25], which results in enhanced thermostability of proteins by lowering entropy change of unfolding [26]. Regarding other structural determinants, formation of extended structures, such as beta sheets, has previously been suggested to be a proteomic signature of thermophilicity [23].

In general, higher content of charged residues is one of the distinctive characteristics of thermophilic proteins [7]. Ratio of charged amino acids to uncharged, more precisely, polar residues [27] has been determined to be the most obvious indicator of hyperthermophilicity. An increase in the amount of hydrogen bonds [28], as well as occurrence of ion-pair networks at a protein surface promotes its thermal stability [29].

Since many acidophilic species are also (hyper)thermoacidophiles, there have been no detailed studies to deduce proteomic adaptation only to lower pH [30]. Cytoplasmic pH of these organisms is close to neutral, thus no specific signatures of adaptation are expected to be found in the cytoplasmic proteins [31]. However, it has been suggested that acidophilic extracellular proteins tend to replace charged residues with neutral polar amino acids [31].

Several studies proposed an increase in charged residue content to be, not only a signature of thermostability, but also of halophilicity [12], [23], [32]. Halophiles have several different strategies to cope with environments with increased salt concentration: by having inorganic ions in the cytoplasm, or by producing organic solutes that maintain osmotic equilibrium [5]. Amino acid substitution of any residue with acidic residue, especially when acidic residues are localized at a protein surface, enhances the protein's solubility in high osmotic environments by extending hydration networks [12], [32].

Certain genomic signatures, such as GC content, and codon usage bias surely contribute to defining amino acid composition. For instance, there are several studies that investigate genomic and proteomic signatures of mesophiles, thermophiles and hyperthermophiles. One of the key observations is that GC content does not have a significant correlation with optimal growth temperatures of prokaryotes [7], [11], nor any mono-nucleotide composition bias is a result of salt preferences of species [12]. Furthermore, amino acid sequence, irrespectively of GC pressure, clearly separates thermophilic from mesophilic species [33].

Primary level of adaptation to higher temperatures, as well as any other environment, is at the amino acid level [11]. However, codon usage bias exists as an independent adaptation on the nucleotide level [11], [34]. For instance, increase in amount of purines (A or AG content) in codons correlates with optimal growth temperatures of prokaryotes, which can be, for instance, due to the codons of IVYWREL [11], IRLG [34], or IVYE [35] amino acids and clearly discriminates (hyper)thermophilic from mesophilic species. The existence of synonymous codon usage bias has also been confirmed for adaptation towards high salt concentrations, and it is salt adaptation specific. Codons of amino acids DEVT are preferred within halophilic species [12].

Our unsupervised selection yielded only features that have already shown important for environmental adaptations. However, we have defined which five features alone are sufficient to describe the variety of environmental adaptation of *Archaea*. These proteomic features appeared to be explanatory enough to distinguish specific adaptations at the level of entire proteome in large datasets.

Furthermore, in our study we did not want to show only connection of abundance of certain amino acids with specific environmental conditions. We aimed at explaining environmental adaptation through quantitative description of physical and biochemical properties of amino acids. Instead of using amino acid composition as a feature, for instance percentage of glutamic acid, we tested additional 7 features such as percentage of polar amino acids, percentage of acidic amino acids, and so forth.

### Phyloecological clusters

In order to compare the proteomes, we computed the distances between each pair of proteomes based on the features subset. The resulting subset containing five proteomic features was used for agglomerative hierarchical clustering.

**Figure 1:**
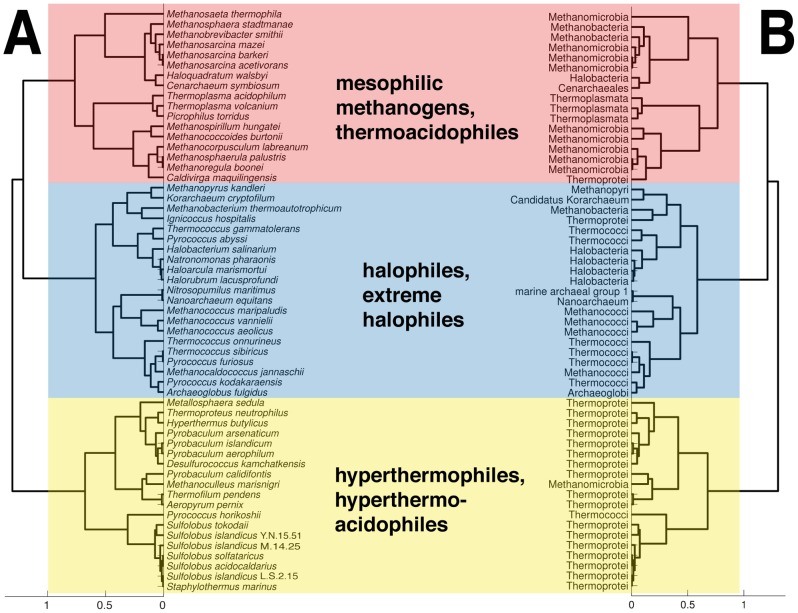
Phyloecological cluster tree of *Archaea*. **A.** The cluster tree was built with average linkage of distances based on correlation metric by using the 5-features subset. Cut-off distance for the formation of clusters was 1.0. The tree is divided into three main phyloecological clusters. The first cluster (highlighted in yellow) is comprised of hyperthermophilic species, non-halophilic or halotolerant, and aerobic hyperthermoacidophiles. The members of the second cluster (highlighted in blue) are halophilic and extremely halophilic *Archaea*, growing in the various temperature ranges (mesophilic, thermophilic or hyperthermophilic values), and conditions of neutral pH or alkaliphilic. The third cluster (highlighted in red) contains mesophilic (mostly methanogens) and thermoacidophilic *Archaea*, non-halophiles or halotolerant. **B.** Leaves of the tree display names of phyla for each species from **A**.

The tree presented in **Fig. 1** was built with correlation distance and average linkage. This tree had the best cophenetic coefficient (c = 0.7291) of all trees built with different combinations of distance metrics and linkage functions.

The strong positive correlation between the cophenetic pairwise distances and proteomic dissimilarities is highly significant (rho = 0.6540, p-value = 1.9599e-195; r = 0.7291, p-value = 7.6776e-265), suggesting that the representative tree illustrates quite well the differences in the proteomic properties.

**Fig. 1** shows that the differences between these five proteomic characteristics divide *Archaea* into three main clusters. It would be superficial only to describe each main cluster with a single adaptation. Namely, while certain archaeal species have been found in specific well-described niches, from our analysis it is possible to suggest additional environmental conditions in which the species could live. As already mentioned, *Archaea* are very diverse phenotypic group, and each archaeon shows several phenotypic adaptations, for instance, *Methanocaldococcus jannaschii* is a deep-sea hyperthermophile, slight halophile, anaerobe and methanogen (details in **Table S1**). Therefore, one can characterize each cluster by several specific environmental adaptations.

The first cluster (highlighted in yellow) is comprised of hyperthermophilic species, non-halophilic or halotolerant (few halophiles), with growth pH ranging from acidophilic to neutrophilic values, and captures majority of aerobic species. The members of the second cluster (highlighted in blue) are halophilic and extremely halophilic *Archaea*, growing in the various temperature ranges (mesophilic, thermophilic or hyperthermophilic values), and conditions of neutral pH or alkaliphilic. The third cluster (highlighted in red) contains mesophiles (mostly mesophilic methanogens) and thermophilic *Archaea*, non-halophiles or halotolerant (few halophiles), growing in acidic or neutral pH conditions.

We suggest such clustering to be termed phyloecological clustering.

By using only the phylogenetic analysis, lineage of Nanoarchaea still remains doubtful [36]. However, our approach emphasizes symbiotic nature of *Nanoarchaeum equitans* and places it in the same cluster with its marine host, *Ignicoccus hospitalis*, based on the similarities of their proteomes. Furthermore, *Pyrococcus horikoshii* (Thermococci) is closer to Thermoprotei, Crenarchaeota than to its own phylogenetic clade. It groups with the majority of hyperthermophiles, in the cluster highlighted in yellow, possibly because its proteomic signature of hyperthermophilicity is stronger than signature of halophilicity. *Methanobacterium thermoautotrophicum* is a halophile with optimal growth temperature higher than in other members of Methanobacteria. Therefore, it belongs to different phyloecological cluster than other members of his clade, it is a part of the “blue” cluster that is characterized by higher salt requirements and temperatures until thermophilic and hyperthermophilic values.

On the other hand, there are two examples in which our method misclassifies species. The proteomic features of mesophilic *Methanoculleus marisnigri* are more similar to thermophiles and hyperthermophiles than to other mesophilic methanogens within the “red” cluster. Also, *Haloquadratum walsbyi* is not sharing the cluster with the rest of extreme halophiles. *H.walsbyi* lives in NaCl-saturated and MgCl_2_ enriched aquatic ecosystems, and has phototrophic growth [37].

### Testing validity of clusters

In order to check if the archaeal phyloecological clustering was obtained as a coincidence, we have performed the clustering of data with permuted values of each feature. The observed clustering did not divide species into phyloecological clusters (data not shown). This implies that randomly assembled set of five actual proteomic features does not contain information on phyloecological properties of the species. Moreover, we computed correlations between proteomic pairwise distances of permuted dataset and proteomic pairwise distances of the original 5-features dataset, and repeated the procedure 10 times. Neither rank nor linear correlations were significant when calculated in all ten cases (p-values>0.15).

### Different clustering technique confirms formation of phyloecological clusters

Archaeal habitats per se do not contain hierarchical information, however, the proteomic features are, indeed, hierarchies. To confirm that hierarchical clustering gives important and robust information, we have applied *k*-means clustering. We predefined the number of clusters, *k* = 3, and as a result we have obtained almost identical clusters, as compared to the ones obtained by hierarchical clustering (see **Fig. S1**; cluster 1 corresponds to the “blue” cluster, cluster 2 resembles the “yellow” cluster, and member of “red” cluster are joined together in cluster 3). There were only two archaea that were clustered differently than by hierarchical clustering. *Methanoculleus marisnigri* now became a member of cluster of methanogenic archaea, which corresponds more accurately to its ecological niche. *Caldivirga maquilingensis*, an anaerobic hyperthermophilic archaeon, joined the cluster comprised of hyperthermophiles. Therefore, it appears that, when applying *k*-means clustering method, this species' preferences towards high temperature and low pH appeared dominant, whereas anaerobic trait prevailed when hierarchical clustering was applied.

Overall, *k*-means clustering generated well-separated clusters – there were no negative silhouette values, and the mean silhouette value was 0.4059. Predefining *k* to a higher number, *k* = 4, 5, 6, or 7, did not improve the quality of clustering (the mean silhouette values were 0.3816, 0.3601, 0.3695 or 0.3608, respectively; also, negative values appeared in these computations).

### Proteomic features as a signature of environmental adaptation

The distribution of several single adaptations within the clusters is given in **Table 1**. The single adaptation that is perhaps the best inferred with this clustering is adaptation to salt; 61% of all halophiles are grouped within the “blue” cluster. This result supports findings of the two previous studies [38], [39], which state that salt requirement determines composition of microbial community better than temperature or other environmental factors.

**Table 1:** Abundances of environmental phenotypes within the clusters.

Adaptation		Abundance of Phenotypes			
	Environmental Phenotype	Yellow Cluster		Blue Cluster	Red Cluster
temperature	Thermophiles	0.364	0.273		0.364
	Hyperthermophiles	**0.538**	**0.423**		0.038
	Mesophiles	0.063	0.250		**0.688**
	Others/Unknown	0.000	0.750		0.250
salt	Halophiles, Extreme Halophiles	0.194	**0.613**		0.194
	Non-halophiles	0.400	0.050		0.550
	Others/Unknown	0.833	0.167		0.000
pH	Acidophiles	**0.636**	0.000		**0.364**
	Neutrophiles	0.250	0.469		0.281
	Others/Unknown	0.286	0.429		0.286
O_2_	Aerobes	**0.571**	0.286		0.143
	Anaerobes	0.250	0.444		0.306
	Others/Unknown	0.286	0.143		0.571
metabolism	Methanogens	0.105	0.316		**0.579**
pressure	Piezophiles	0.143	**0.857**		0.000

The table presents abundances of specific environmental phenotypes. It is hard to infer adaptation to a single condition as the dominant phenotype for each main cluster. Perhaps only one environmental phenotype could be emphasized, and that is halophilicity, which is dominant within the “blue” cluster. The bolded numbers depict phenotypes that are prevailing within the respective clusters, however, they are mainly coupled with another adaptation; e.g. mesophilic methanogens in the “red” cluster or hyperthermoacidophiles in the “yellow” cluster.

Since the vast majority of halophilic species occupies the “blue” cluster, we considered possibilities that could explain why few halophiles are members of the other two clusters. Our hypothesis was that some other adaptation could have stronger proteomic signal than salt adaptation in this specific case. All halophiles that are not in the “blue” cluster and have hyperthermophilic adaptation are members of the “yellow” cluster, and all halophiles that are meso- or thermophilic are in the “red” cluster; which corresponds to the previously described characteristics of these two clusters. This suggests that temperature adaptation signature has primacy over salt adaptation signature in the proteomes of the halophiles outside of the “blue” cluster.

**Figure 2:**
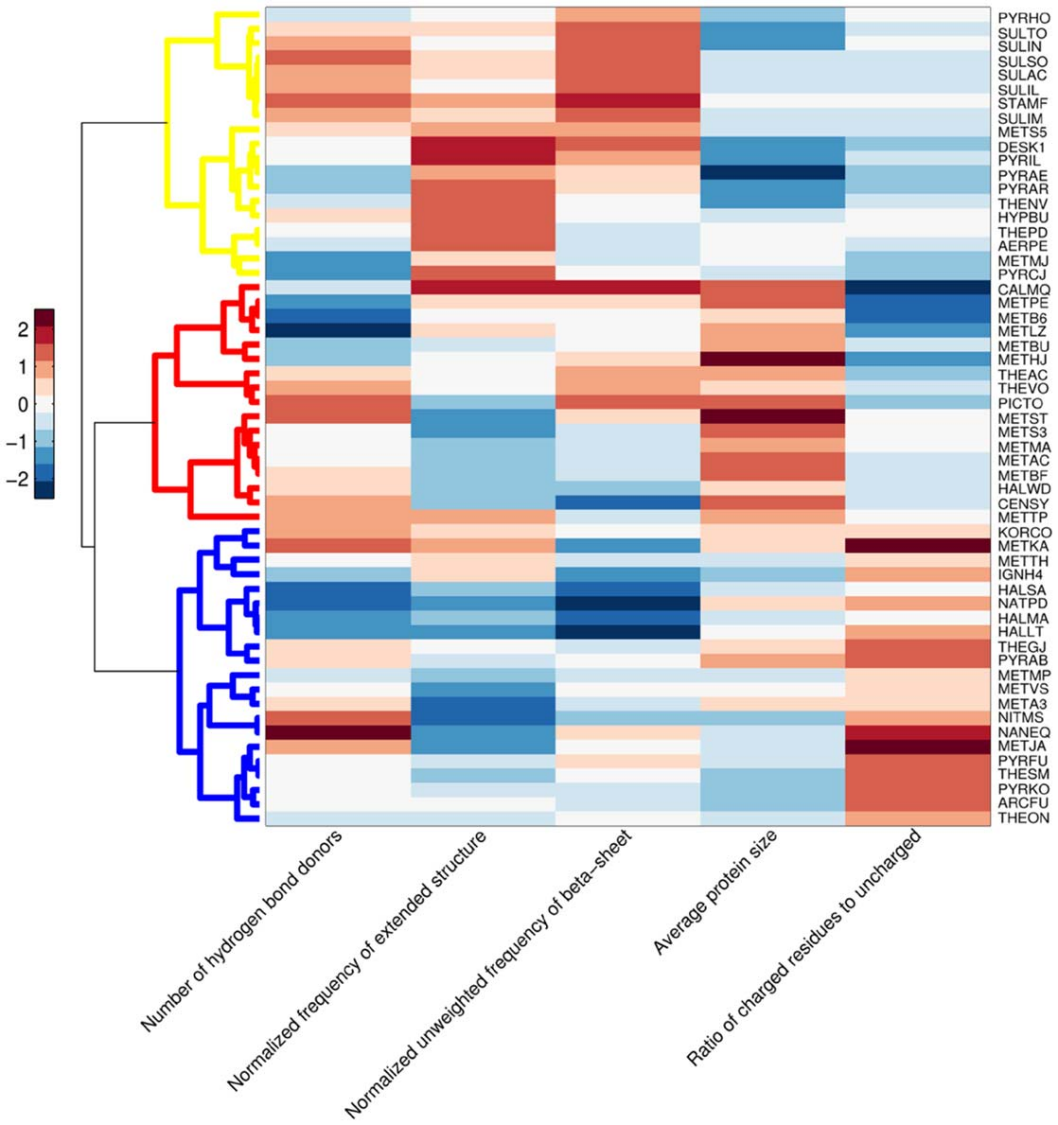
Heat map visualization of archaeal proteomic properties across phyloecological clusters. In this heat map, the rows correspond to each species and the columns correspond to the proteomic features; therefore, each row depicts the subset of 5 proteomic features per given archaeon. Normalized values of each feature are associated with the colors in the heat map as shown on the legend on the left side. The cluster tree was built with correlation metric and average linkage, same as in **Fig. 1**. Three main phyloecological clusters are highlighted with three different colors of the branches (yellow: hyperthermophiles and hyperthermoacidophiles; blue: halophiles and extreme halophiles; red: mesophilic methanogens and thermoacidophiles).

The heat map in **Fig. 2** illustrates the values of five proteomic features across different archaeal species, and, in the same time, displays clustering of *Archaea* based on their phyloecological characteristics. Coloring that is in accordance with normalized values of the features depicts variation in protein properties throughout the different archaeal habitats. In this figure, the quantitative differences in each proteomic property are noticeable. For instance, METJA (hyperthermophile and halophile) and METKA (hyperthermophilic slight halophile) have the highest number of hydrogen bond donors, followed by NANEQ (hyperthermophilic halophile); moreover, they exhibit an increase in the amount of charged residues. In general, mostly hyperthermophilic halophiles have the highest ratio of charged residues over uncharged, followed by halophiles, and then hyperthermophiles. The lowest ratio of charged amino acids to uncharged mostly have (hyper)thermoacidophiles and mesophilic methanogens. Furthermore, it can be seen that hyperthermophiles have smaller average protein size than mesophilic methanogens. The “yellow” cluster containing hyperthermophiles and hyperthermoacidophiles also displays an increase in occurrences of extended structures and beta-sheets.

### Subset of five proteomic features contains more information than the entire dataset

We have also assessed if the tree built on the 5-features subset represents the proteomic data more accurately than the tree built on the entire set of 58 features. Therefore, we performed hierarchical clustering on the entire set by using the same parameters: correlation metric and average linkage. First, the cophenetic coefficient was worse (c = 0.7014) for this tree. Second, the correlation between the cophenetic distances and proteomic pairwise dissimilarities was not as strong as for the tree built upon the 5-features subset (rho = 0.5855, p-value = 1.6878e-147; r = 0.7014, p-value = 1.2228e-236). This means that the tree built with the entire dataset does not represent the proteomic distances as faithfully as the 5-features subset, and that the 5-features tree has more power to discriminate the phyloecological clusters. Third, we asked how much these two datasets (two pairwise matrices) are actually similar. The correlation between dissimilarities computed between 5 features and dissimilarities computed between 58 features was strong and positive (rho = 0.7245, p-value = 0; r = 0.7382, p-value = 7.8547e-275), suggesting that there is sufficient amount of information already in the 5-features subset.

To conclude, the tree built by using the 5-features subset shows more precisely the differences between the species, and at the same time still captures the significant portion of total proteomic information.

### Assessment of the proteomic features' variation within habitat space

We have tested if the subset of five proteomic features has consistent values when analyzed in less dimensions of the multiple habitat-space, for instance, if hyperthermophilic/mesophilic halophiles show similar proteomic feature signals for temperature adaptation as hyperthermophilic/mesophilic non-halophiles. For this purpose, we applied Mann-Whitney test and Kolmogorov-Smirnov test (for two independent samples) to determine if medians and distributions of the proteomic features are the same across one environmental adaptation irrespective of the second environmental adaptation.

The proteomic signature of mesophiles is consistent, regardless if the mesophiles are halophilic or non-halophilic (**Table S1**, assessment of variation). When analyzing only hyperthermophiles, three proteomic features are significantly different between hyperthermophilic halophiles and hyperthermophilic non-halophiles. Normalized frequencies of extended structure and beta-sheet are significantly higher in non-halophiles than in the subset of hyperthermophilic halophiles. Ratio of charged amino acids to uncharged is also significantly higher in halophilic hyperthermophiles than in non-halophilic hyperthermophiles. On the other hand, the feature describing average protein size has almost the same value between the groups of hyperthermophilic halophiles and hyperthermophilic non-halophiles (**Table S1**, assessment of variation). This feature has been uniquely attributed to temperature adaptation, where hyperthermophilic proteins tend to be significantly shorter than mesophilic proteins [7], [25], [26], and it is thus possible that does not capture differences in salt adaptation.

Other proteomic features most likely capture synergistic or antagonistic effect of multiple adaptations. Nonetheless, as discussed above, it is very difficult to claim that the proteomic signal is attributed to a single, particular adaptation.

### Phyloecological tree vs. phylogenetic tree

We sought to compare the clustering results obtained herein with the distances in the tree obtained by phylogenetic tools.

We computed correlations between pairwise phylogenetic distances generated by Jukes-Cantor model and pairwise proteomic distances calculated in this study by using correlation metric. The correlation between phylogenetic distances and proteomic distances was significant and weakly positive (rho = 0.2497, p-value = 4.1240e-24; r = 0.0774, p-value = 0.002). Moreover, we have computed Mantel correlation, another method used to correlate pairwise distances generated by two different approaches. Mantel correlation between pairwise phylogenetic and proteomic distances revealed a correlation coefficient of 0.2497 and p-value of 0.0.

This indicates that the phyloecological tree does not dominantly represent the phylogenetic relationships between the species, and likewise captures their common environmental adaptations (aspects of archaeal evolution and phylogeny are discussed in [36], [40]–[42]).

Herein, we have focused on physicochemical properties of the proteome: such properties are a consequence of environmental adaptation of species to their environment, but not only the adaptation that happened recently in evolutionary history. The reconstruction of evolutionary history relies on available DNA or specific protein sequences and, nowadays, many mathematical models tend to explain evolutionary relatedness among species. Since it seems unlikely that a single phylogenetic framework or sequence standard, such as 16S rRNA, will be sufficient for classifying all prokaryotic organisms, it is possible to suggest a possibility to explain evolutionary and environmental relations based on physicochemical properties of the proteome. When taking the whole proteome into account, one does not discard the influence of horizontal gene transfer and, also, avoids trees built on concatenated proteins that probably follow different models of evolution. Thus, it could be achievable to describe the properties of a proteome in the framework of the environmental niche in which it has arisen as a consequence of adaptive evolution. Since species that share older evolutionary history (and also adaptations that happened a long time ago in evolution) are phylogenetically related, there will always be similarities between phylogenetic and phyloecological trees.

Phylogenetic trees can be built by using various methods: 16S rRNA sequences, concatenated protein sequences, whole-genome comparison based on gene content, genome conservation methods, and so forth (examples in [36], [40], [43]). In this study, we have put emphasis on the proteome sequence because it is a primary indicator of certain environmental adaptations [11]; however, we aimed at avoiding the usage of simple sequence alignment procedures. All proteins, regardless of having deleted or inserted parts of sequences in comparison to their orthologs, needed to be adapted on existing conditions of a habitat. In the study of Tekaia and Yeramian, amino acid composition of proteomes showed a straightforward discrimination of species according to unified lifestyle-phylogenetic classes [10]. Correspondingly, our method allows us to investigate the signatures of both phylogenetic history and ecological adaptation, and therefore we named this analysis phyloecological clustering.

### All features – except average protein size – exhibit significant phylogenetic signal

We have used Generalized linear mixed model with Markov chain Monte Carlo estimation [44] to infer influence of phylogenetic relationships in the formation of the phyloecological clusters. We computed how much of the variance of each proteomic feature is explained by phylogenetic signal, and which fraction carries the signature of the ecological niche. The only feature that has not shown significant correlation with the phylogeny is the one describing average protein size, the only feature that does not depend on amino acid composition of the proteome. In that case, phylogenetic distance explained only 28.4% of its total variance, whereas 71.6% of the variance was due to the affiliation with the phyloecological clusters (**Table S1**, MCMC variance). Phylogenetic relationships explained more than 98% of the total variance of the remaining four features, which is due to the fact that these features were deduced from amino acid composition of the proteome.

Moreover, we used the same model to infer the extent to which each feature contributes to the definition of clusters, given their phylogenetic signal. When corrected for phylogenetic relationships, the features still contributed significantly to separation of the phyloecological clusters (**Table S1**, MCMC). This analysis confirmed the results discussed above: biochemical and physical nature of each feature reveals clear environmental signals. The feature describing average protein size has appeared important for separating mesophilic species in the “red” cluster from thermophiles and hyperthermophiles. Ratio of charged amino acids to uncharged, as well as frequency of beta-sheet proved to be significant for gathering halophiles in the “blue” cluster, separately from non-halophilic hyperthermophiles and mesophilic methanogens. Frequency of extended structure seemed to be important for distinguishing between the members of the “yellow” cluster and the “red” cluster, possibly due to different values for salt concentration, pH and temperature in the niches that are characterized by these two clusters. The feature describing number of hydrogen bond donors contributes the least to the definition of the phyloecological clusters.

### Predictive power of the phyloecological clustering

**Figure 3:**
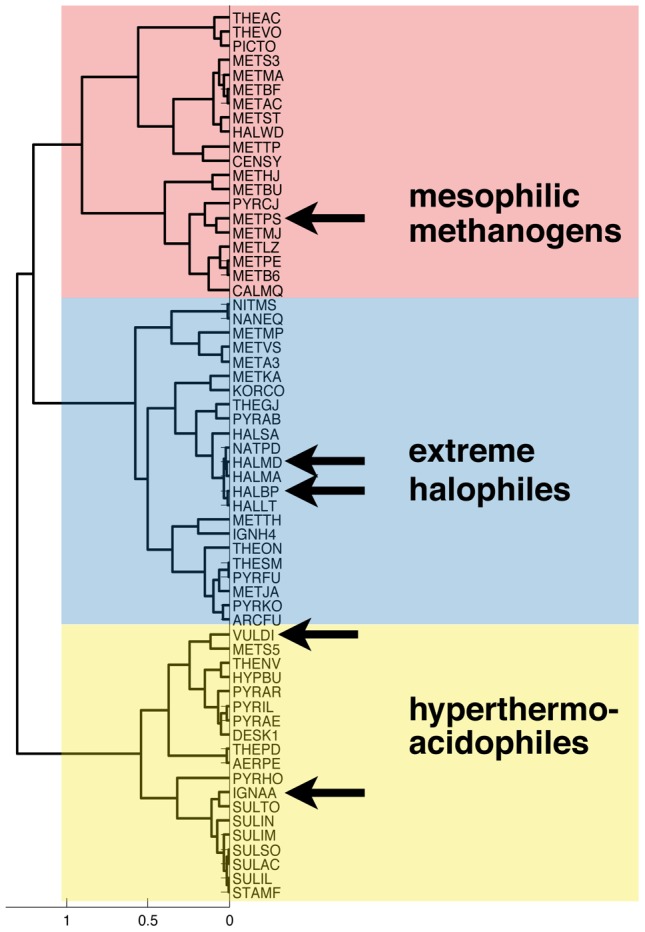
Assessing predictive power of phyloecological clustering. We added five new species to the dataset and preformed hierarchical clustering with the established parameters. The new species became a part of the clusters defined with similar environmental conditions as it is found in their respective niches. One archaeon joined the “red” cluster with other mesophilic methanogens; two species merged with the members of halophilic ”blue” cluster; and two hyperthermoacidophilic organisms became members of the corresponding “yellow” cluster.

We were interested if our method is able to predict environments of species whose proteomes were not included in the analysis described herein. We added to our dataset five species whose proteomes became available after the beginning of our study [45], and performed clustering on 62 species by using 5-features subset. We found that our phyloecological tree puts these species in the clusters corresponding to their environmental niche (**Fig. 3**). METPS, a mesophilic methanogen, became a member of the “red” cluster. HALBP and HALMD are extreme halophiles and, therefore, they merged with other halophilic species in the “blue” cluster. VULDI, hyperthermoacidophile, and IGNAA, hyperthermophilic moderate acidophile, both joined the corresponding “yellow” cluster.

### Including extremophilic bacteria in the analysis

Most of the recent studies focus on adaptations of prokaryotic species [11], and some of the analyses also include a certain number of eukaryotes [7]–[10]. However, it has been argued that bacterial and archaeal species show clear differences at the proteomic level [23]. We have included features of several extremophilic bacteria (halophiles, acidophiles, hyperthermophiles, etc.) in the clustering, which changed significantly the topology and interpretation of the cluster tree shown in **Fig. 1** (data not shown). One of the explanations for the variability of the cluster tree could be that the features distinguishing *Bacteria* from *Archaea* largely influence the outcome of clustering [23]. Although adaptation to diverse environmental conditions should be universal, we focused only on *Archaea* to reduce any possible domain of life bias.

## Conclusions and Future Directions

The approach of recursive search for uncorrelated features has allowed us to identify five non-redundant physicochemical properties of the archaeal proteomes. Clustering upon these five features led to grouping of *Archaea* according to their environmental adaptations and contributed to better understanding of relations within this domain of life. Therefore, we applied two different procedures, both of which are unsupervised. In general, unsupervised methods have been proved quite useful to deducing from large datasets. In the recent study, unsupervised binning was used to reveal phylogenetic signals from metagenomic sequences [46]. Assuming that entire metagenome was imposed to same environmental pressure, resulting dis(similarities) within sequencing data can be attributed mostly to species origin.

Similar to sequence binning that sorts contigs and scaffolds of multiple species in a metagenome [46], our method can be described as proteome binning based on proteome sequence features that results in bins of proteomes with common environmental adaptation. In addition, we may propose an approach that would allow for binning of individual protein sequences based on their environmental adaptations. In such a procedure, a non-redundant subset of protein properties can be applied to cluster sequences according to their physicochemical properties within ecological niches and discussed in the framework of protein function adaptation to environmental conditions.

Further availability of sequenced proteomes will certainly increase the quality of such analysis of archaeal phyloecological clusters. For example, our current dataset comprises 7 species that live in the deep-sea habitats (piezophiles or barophiles [47]). Our analysis puts 6 species in the “blue” cluster (*Methanococcus aeolicus*, *M. jannaschii*, *Methanopyrus kandleri*, *Pyrococcus abyssi*, *Thermococcus gammatolerans*, *Thermococcus onnurineus*). Analyzing more piezophiles with available proteomes could improve statistical relevance of categorizing species with such adaptation. Also, in future one could ask, if there are additional habitats where certain species could be found, and which *Archaea* could co-exist with it.

As recently reviewed in Zaneveld *et al.* [48], combining existing tools in phylogeny with ”-omics” approaches is necessary to address questions of microbial adaptation to habitat. Therefore, we propose this type of phyloecological analysis as a source of additional information to the basic phylogenetic methods.

Considering how fast new proteome sequences are becoming available, we propose re-analysis of the few-fold more archaeal proteomes by using our method of phyloecological clustering. It would be interesting to evaluate the results on larger datasets. Adding to the list more archaeal species with different habitats will further estimate the robustness of this method; the same principle has been already applied to different methods of analysis of proteomic signatures [27].

## Methods

### Archaeal environments and the proteomes

We have analyzed a total of 57 archaeal species: all archaeal proteomes available at the beginning of this study with accurate description of their environmental niche. The proteome sequences were collected from the HAMAP database [45] UniProtKB/Swiss-Prot Release 57.7 of 01-Sep-2009, which is based on manual annotation of the proteins from the closed genomes. We have described each proteome with 58 features in total: 50 amino acid attributes as suggested by Atchley *et al.* [22], including 8 more features defined herein and listed in **Table S1**. Each proteomic feature was based on amino acid composition of the proteome and gave information about physical and chemical characteristics of the proteome (e.g. negative charge, hydrophobicity, propensity of secondary structures, etc.). Additionally, we included a feature that describes the average length of a protein within the proteome.

The proteomic features were computed as follows. First, every archaeal protein was described with 58 features based on its amino acid composition. Finally, all features were computed for every protein within a proteome, and then averaged over all proteins in the proteome. A contribution of an amino acid unique for methanogens, pyrrolysine [49], was not included in the following analysis, except for the feature explaining average protein size.

Furthermore, we harvested databases and other literature sources to retrieve information about physicochemical properties of the environment where each organism optimally lives (**Table S1**). We have organized this information into the database available at http://cbb.medils.hr/penbase.

### Unsupervised feature selection

We performed unsupervised feature selection by recursively discarding proteomic features correlated more than 0.5 (sample correlation).

The procedure was as follows:

i) correlation coefficients were computed between each pair of features,
ii) a pair of features with the highest absolute value of the correlation coefficient was noted. Between these two features, the one that is discarded is the one with the highest correlation coefficient towards a third feature. This way, the user only determines the threshold: our final feature subset is comprised of features correlated among themselves less than 0.5.

Herein described computational procedures, as well as all analyses mentioned in the further text were performed in Matlab, version MATLAB_R2010b (the scripts are assembled in **File S1**).

### Clustering of the subset

We calculated the distances between each pair of proteomes based on the features subset and performed agglomerative hierarchical clustering (see **File S1**).

The input matrix for hierarchical clustering consisted of rows, corresponding to the archaeal species, and columns, corresponding to the proteomic features. First, we normalized the values of the proteomic features (values of each proteomic feature now had zero mean and unity variance). Then we applied four distance measures (Euclidean distance, correlation distance, cosine similarity, city block distance) to compute pairwise distances between species based on the features subset. Two linkage methods (unweighted average distance (UPGMA), weighted average distance (WPGMA)) were applied on the pairwise distances in order to build cluster trees. Thus, each cluster tree was built by applying one distance metric and one linkage method. The goodness of each clustering was evaluated by computing the cophenetic correlation coefficient. Cutting the branches below defined distance led to formation of clusters from the tree. The tree that had the best cophenetic coefficient was chosen to be a representative result. In order to assess how the representative tree illustrates the differences in the proteomic features, we computed correlations between pairwise distances (i.e. proteomic dissimilarities) and cophenetic distances.

Matlab functions *pdist*, *linkage*, *cophenet* and *dendrogram* (Statistics Toolbox) were used to build the cluster trees.

The heat map was created for the same representative tree to illustrate the quantitative differences between proteomic features across different archaeal clusters (see **File S1**). Matlab function *clustergram*, which is a part of Bioinformatics Toolbox, was used to create the heat map.

### Statistical evaluations of clustering

The procedures were evaluated statistically by using Pearson's linear (calculating r) and Spearman's rank (calculating rho) correlations.

The validity of formed clusters was tested as follows. For each feature (each column) we permuted the values within the feature. Therefore, each species now had different values of its proteomic features. We applied clustering on the resulting dataset with permuted values of each feature (see **File S1**).

### *k*-means clustering of the subset

We applied *k*-means clustering on the subset of the proteomic features (Matlab function *kmeans*, Statistics Toolbox; correlation distance metric, initial cluster position selected at random, and repeating the procedure 8 times, see **File S1**). This type of clustering uses a point-assignment algorithm, which is a different algorithm than the one used for hierarchical clustering. We used the hierarchical cluster tree in order to predefine number of clusters *k*, however, we also performed clustering on different values of *k*. Mean silhouette value was computed to evaluate validity of the clusters, i.e. to show how similar are the members within each cluster.

### Clustering of the entire dataset

We performed hierarchical clustering on the entire set of proteomic features by using the same parameters as for the representative tree. We aimed to compare the tree built on the entire set of 58 features with the tree built on the features subset and computed: (1) cophenetic coefficient of the tree built upon the entire set, (2) linear and rank correlations between the cophenetic distances and dissimilarities for the tree based on the entire set (3) linear and rank correlations between pairwise distances computed from the features subset and pairwise distances computed from the complete set of the features.

### Variation of proteomic features

In order to assess variation of proteomic features within multidimensional habitat space, we divided the dataset into 2 subsets: halophiles (31 species) and non-halophiles (20 species). We further divided each of these 2 subsets into three groups depending on temperature preferences of the species: hyperthermophiles, thermophiles and mesophiles. For each group coming from the different subsets we performed Mann-Whitney test (for two independent samples) on each proteomic feature to determine if the medians of the proteomic features of two subsets are the same. We also performed Kolmogorov-Smirnov test (for two independent samples) to determine if the proteomic features of two subsets come from the same distribution (see **Table S1**). Both of these tests were performed by using SPSS statistical software, version 19.

### Phylogenetic tree

The 16S rRNA sequences of all *Archaea* were gathered from NCBI database. The phylogenetic distances were computed by using Jukes-Cantor model on the multiple alignment of the 16S rRNA sequences (see **File S1**). Jukes-Cantor pairwise distances were then used to build the tree of archaeal species with the linkage method UPGMA. The phylogenetic tree was finally saved as Newick-formatted file. Matlab functions *fastaread*, *multialign*, *seqpdist, seqlinkage* and *phytreewrite* (Bioinformatics Toolbox) were used for this purpose.

Linear and rank correlation coefficients were then computed to assess the correlation between the phylogenetic pairwise distances (Jukes-Cantor model) and the proteomic pairwise distances.

Mantel test was also used to test for correlations between the pairwise phylogenetic and proteomic distances (Matlab custom script [50]; 250′000 iterations). This test is usually used in ecology to assess, for example, the relation between pairwise geographical and genetic distances [51].

### Phylogenetic comparative analysis

We have used the Markov chain Monte Carlo (MCMC) approach to fit Generalized Linear Mixed Model to the subset of the normalized proteomic features. Models were run for 200′000 iterations including a burn-in of 30′000 iterations, and a thinning interval of 150 (taking into account only every 150th iteration, which reduces the autocorrelation between iterations). We performed this analysis by using ‘MCMCglmm’ in R, version 2.10.1 [44] (see **File S1**). Flat priors were set according to [52], and the lower and upper modes of 95% highest posterior densities were parameter estimates accessible in **Table S1**, MCMC. This method has certain advantages over classical Independent Contrasts – for instance, it allows analysis of traits that are not normally distributed, and supports multi-response models where the responses can follow different distributions [53].

### Predictive power of the cluster tree

We have tested if the clusters formed on 57 species can predict the environmental conditions of several archaeal species whose proteomes were not included in the feature selection, and cluster formation and analysis. We have randomly selected five new species: *Halogeometricum borinquense* (HALBP), *Halomicrobium mukohataei* (HALMD), *Ignisphaera aggregans* (IGNAA), *Methanocella paludicola* (METPS) and *Vulcanisaeta distributa* (VULDI), which were included in HAMAP database after we had started this project [45]. We associated their positions in the tree with the corresponding environment they were originally isolated from.

### Testing universality of environmental adaptations

We included five extremophilic bacteria in the clustering based on the features subset: *Acidothermus cellulolyticus* (aerobic thermoacidophile), *Acidiphilium cryptum* (acidophile), *Aquifex aeolicus* (halophilic hyperthermophile), *Salinibacter ruber* (extremely halophilic aerobe) and *Thermus thermophilus* (thermophile). Our aim was to observe whether cluster tree retains its environmental clusters or it changes topology due to phylogenetic differences between *Bacteria* and *Archaea*.

## Supporting Information

Figure S1 **The result of** ***k*** **-means clustering.** The clusters made with this method confirm the result of hierarchical clustering and presence of phyloecological signal in the proteomic features.
(TIFF)

Table S1 Information on the archaeal species. The table provides the list of 57 archaeal species analyzed in this study; the links to HAMAP webpage, http://us.expasy.org/sprot/hamap/, where the proteomes have been described; the description of the ecological niches gathered from the original papers or databases; archaeal taxonomy according to NCBI taxonomy database, http://www.ncbi.nlm.nih.gov/taxonomy; and details of some analyses done in this study.
(XLS)

File S1 The Matlab and R scripts used in this study.
(PDF)
