# Supplementary figures and images for: Proteomic Properties Reveal Phyloecological Clusters of Archaea
Source: PLoS One. 2012 Oct 25;7(10):e48231. doi: 10.1371/journal.pone.0048231 (PMC3485053; doi:10.1371/journal.pone.0048231)

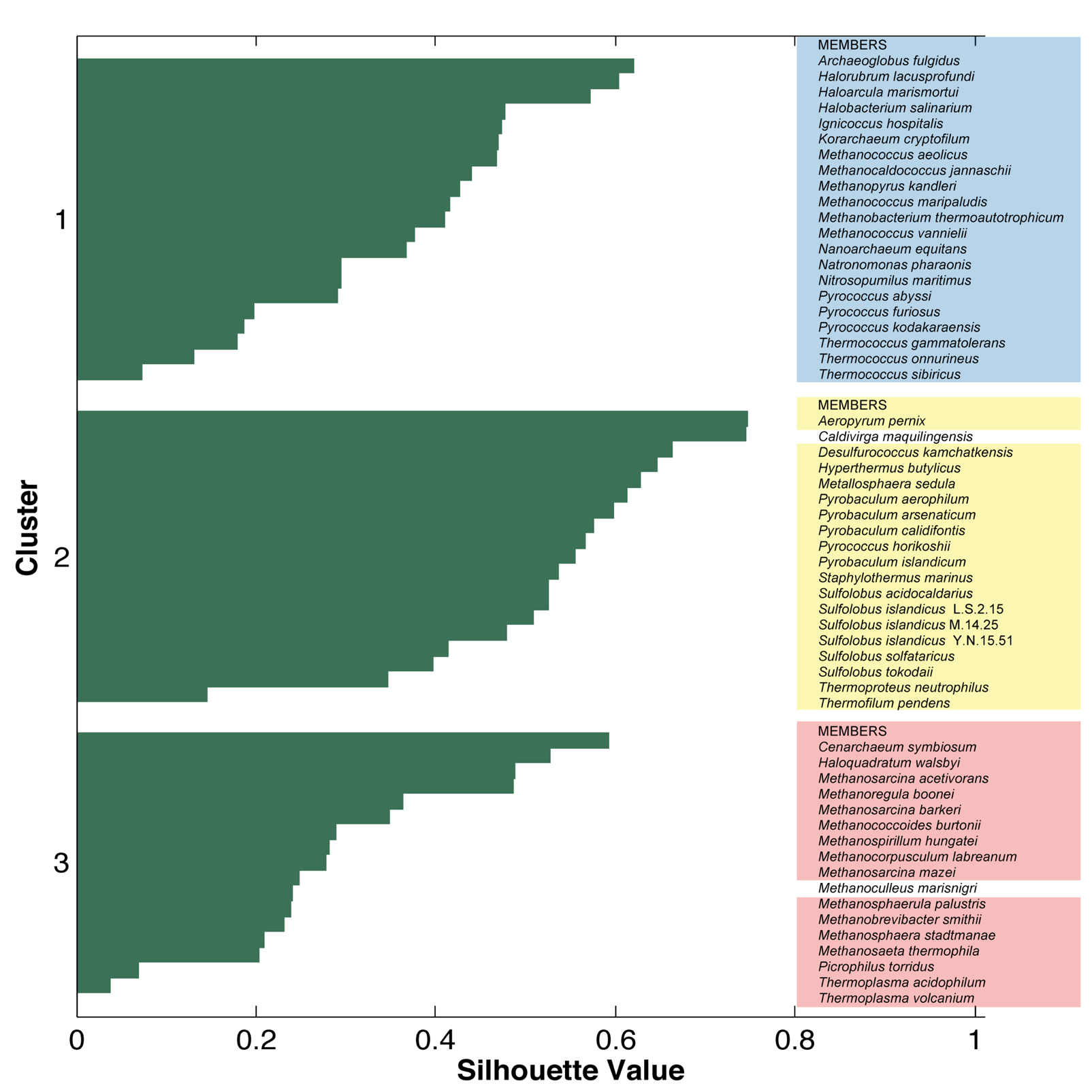

Supplement: Figure S1 — The result of k -means clustering. The clusters made with this method confirm the result of hierarchical clustering and presence of phyloecological signal in the proteomic features. (TIFF) [file pone.0048231.s001.tif]
