# Supplementary material for: Proteomic Properties Reveal Phyloecological Clusters of Archaea
Source: PLoS One. 2012 Oct 25;7(10):e48231. doi: 10.1371/journal.pone.0048231 (PMC3485053; doi:10.1371/journal.pone.0048231)
Supplement: File S1 — The Matlab and R scripts used in this study. (PDF) [file pone.0048231.s003.pdf]

% Matlab scripts

% A_recursive_uncorrelated

```
% Nela Nikolic, 2011

%% Generates features subset

% Load an input matrix: matrix 57 species x 58 descriptors
% The input matrix for hierarchical clustering is consistent of rows, corresponding to archaeal species,
% and columns, corresponding to proteomic features.

load ArchaealMatrix57x58.mat;
Mat=ArchaealMatrix57x58;

DescriptorsList=importdata('DescriptorsList.txt');

max_value=1;

% Normalize matrix - zero mean and unity variance
Mattrans=transpose(Mat);
Matnorm=mapstd(Mattrans);
Matfinal=transpose(Matnorm);

Matfirst=Matfinal;
originalnumber=size(Matfirst,2);

while(max_value>0.5)
        species=size(Matfinal,1);
        numberofdescriptors=size(Matfinal,2);
        list_i=[];
        list_j=[];

        Matcorr=corrcoef (Matfinal);
        Matcorr_zeros=Matcorr;

        for i=1:numberofdescriptors
                if(Matcorr_zeros(i,i)==1)
                        Matcorr_zeros(i,i)=0;
                end
        end

        abs_Matcorr_zeros=abs(Matcorr_zeros);

        % Find maxima of all columns
        [column_maxima, column_ind_max]=max(abs_Matcorr_zeros);
        max_value=max(column_maxima);

        z=0;

        for i=1:numberofdescriptors
```

```
                for j=1:numberofdescriptors
                        temp=abs_Matcorr_zeros(i,j);
                        if(temp==max_value)
                                z=z+1;
                                list_i(z)=i;
                                list_j(z)=j;
                        end
                end
        end


        first_max_i=list_i(1);
        first_max_j=list_j(1);
        second_max_i=list_i(2);
        second_max_j=list_j(2);

        Mat_sorted=sort(abs_Matcorr_zeros,1,'descend');


        if(Mat_sorted(2,first_max_j)>Mat_sorted(2,second_max_j))
                discard=first_max_j;
        elseif (Mat_sorted(2,first_max_j)<Mat_sorted(2,second_max_j))
                discard=second_max_j;
        else
                disp(sprintf('critical point - maximum: %d\n',column_maxima))
        end

        Matfinal(:,discard)=[];
        Mat_recursive=Matfinal;
end


% Compare Mat_recursive and Matfirst

finalset_descriptors=size(Mat_recursive,2);
sub=0;
subset=[];

for f=1:finalset_descriptors
        for m=1:originalnumber
                if (Mat_recursive(:,f)==Matfirst(:,m))
                        sub=sub+1;
                        subset(sub)=m;
                end
        end
end

subset
Descriptors_in_subset=DescriptorsList(subset)

%%%%%%%%%%%%%%%%%%%%%%%%%
```

% B_cluster_recursive_results

```
%% Performs hierarchical clustering

% Load a matrix species x descriptors

load ArchaealMatrix57x58.mat;
Mat=ArchaealMatrix57x58;

T=importdata('ArchaeaList.txt');
%load taxa.mat;
%T=taxa;

% Subset that is the result of A script ::  5    39    40    50    55
RecursiveResults=[Mat(:,5),Mat(:,39),Mat(:,40),Mat(:,50),Mat(:,55)];

% Normalize matrix - zero mean and unity variance
Mattrans=transpose(RecursiveResults);
Matnorm=mapstd(Mattrans);
Matfinal=transpose(Matnorm);

%dist = euclidean, cityblock, cosine, correlation
Y=pdist(Matfinal,‘correlation’);

%linkage = average, weighted
Z=linkage(Y,‘average’);

% Verifying the cluster tree
% Computing correlations between the dissimilarities and the cophenetic distances

[c,D]=cophenet(Z,Y);
c
[RHO,PVAL]=corr(Y',D’,'type','spearman')
[R2,p]=corr(Y',D','type','pearson')

figure(1), [tree,S]=dendrogram(Z,0,‘labels’,T,‘Orientation’,‘left’,'colorthreshold',1.0);
set(tree,'LineWidth',3);
```

%%%%%%%%%%%%%%%%%%%%%%%%%%%%

% **C_clustergram**

```
%% Generates heat map

%load a matrix species x descriptors
load ArchaealMatrix57x58.mat;
Mat=ArchaealMatrix57x58;

T=importdata('HAMAP_list.txt');

Descriptors=importdata('DescriptorsList.txt');

% Subset that is the result of A script ::  5    39    40    50    55
RecursiveResults=[Mat(:,5),Mat(:,39),Mat(:,40),Mat(:,50),Mat(:,55)];
FeatsSubset=[Descriptors(5,:),Descriptors(39,:),Descriptors(40,:),Descriptors(50,:),Descriptors(55,:)];

% Normalize matrix - zero mean and unity variance
Mattrans=transpose(RecursiveResults);
Matnorm=mapstd(Mattrans);
Matfinal=transpose(Matnorm);


fig=clustergram(Matfinal,'Standardize','None','ColumnLabels',FeatsSubset,'RowLabels',T,'Cluster',
'Column','RowPDist','correlation','linkage','average','ColorMap','redbluecmap','Dendrogram',
{'colorthreshold', 1.0 },'ColumnLabelsRotate',45);

rendfig=plot(fig)
```

%%%%%%%%%%%%%%%%%%%%%%%%%%%%

% **D_control_by_permuting**

```
%% Permutes values of each feature and performs clustering.

% Load a matrix species x descriptors

load ArchaealMatrix57x58.mat;
Mat=ArchaealMatrix57x58;

T=importdata('ArchaeaList.txt');

% Subset that is the result of A script ::  5    39    40    50    55
RecursiveResults=[Mat(:,5),Mat(:,39),Mat(:,40),Mat(:,50),Mat(:,55)];

% Normalize matrix - zero mean and unity variance
Mattrans=transpose(RecursiveResults);
Matnorm=mapstd(Mattrans);
Matfinalreal=transpose(Matnorm);

Matfinalpermute=Matfinalreal;

numberofdescriptors=5;
for j=1:numberofdescriptors
        Mixing=Matfinalpermute(:,j);
        [foo,ind]=sort(rand(length(Mixing),1));
        Matfinalpermute(:,j)=Mixing(ind);
end

Matfinal=Matfinalpermute;

Y=pdist(Matfinal,'correlation');
Z=linkage(Y,'average');

% Verifying the cluster tree
c=cophenet(Z,Y)

figure(1), [tree,S]=dendrogram(Z,0,'labels',T,'Orientation','left','colorthreshold',1.0);
set(tree,'LineWidth',2);

% Load a file containing pairwise proteomic distances
load pdist_clustering.mat;
clusters=pdist_clustering;

[RHO,PVAL]=corr(Y', clusters', 'type', 'spearman')
[R2,p]=corr(Y', clusters', 'type', 'pearson')
```

%%%%%%%%%%%%%%%%%%%%%%%%%%%

% E_treeBuilder_Archaea

```
%% Computes phylogenetic distances

% Load a file containing sequences in fasta format
rRNA=fastaread('Archaeal_16SrRNA_HAMAP_sorted_HAMAPcode.txt');

T=importdata ('HAMAP_list.txt');

% Align multiple sequences
aligned_rRNA=multialign(rRNA,'ScoringMatrix','NUC44');

% Compute distance matrix by using Jukes-Cantor method

phyla=seqpdist(aligned_rRNA,'Method','Jukes-Cantor','Alphabet','NT');

% Load the file containing pairwise proteomic distances
load pdist_clustering.mat;
clusters=pdist_clustering;

[RHO,PVAL]=corr (phyla', clusters', 'type', 'spearman')
[R2,p]=corr(phyla', clusters', 'type', 'pearson')

tree=seqlinkage(phyla,'average',T);

h=plot(tree,'orient','left');
xlabel('Phylogenetic distance')
title('Distance tree of Archaea using Jukes-Cantor model');

% Write phylogenetic tree object to Newick-formatted file

phytreewrite('archaeatree.tree',tree)
```

%%%%%%%%%%%%%%%%%%%%%%%%%%%%%%%%

% F_kmeans_Archaea

```
%% Performs k-means clustering

% Load a matrix species x descriptors

load ArchaealMatrix57x58.mat;
Mat=ArchaealMatrix57x58;

T=importdata('ArchaeaList.txt');

% Subset that is the result of A script ::  5    39    40    50    55
RecursiveResults=[Mat(:,5),Mat(:,39),Mat(:,40),Mat(:,50),Mat(:,55)];


% Normalize matrix - zero mean and unity variance
Mattrans=transpose(RecursiveResults);
Matnorm=mapstd(Mattrans);
Matfinal=transpose(Matnorm);

k=3;

% Distance metric can be sqEuclidean, cityblock, cosine, correlation (Hamming suitable for binary data)
[idx,c]=kmeans(Matfinal, k, 'distance', 'correlation', 'start', 'sample', 'replicates', 8);


figure(1)
[silh,h]=silhouette(Matfinal,idx);
xlabel('Silhouette Value')
ylabel('Cluster')

mean(silh)

for i=1:k
   clust=find(idx==i);
   T(clust)
end
```

%%%%%%%%%%%%%%%%%%%%%%%%%%%%%%
%%%%%%%%%%%%%%%%%%%%%%%%%%%%%%

########
########

R script

MCMC-based algorithm

Rolf Kümmerli, 2012
MCMCglmm Course Notes by Jarrod Hadfield, available online

```
install.packages("ape")
install.packages("MCMCglmm")
library("ape")
library("MCMCglmm")

# Load a tree as Newick-formatted file
atree<-read.tree(archaeatree.txt")

# Load a table containing following columns:
# animal, cluster, feat1, feat2, feat3, feat4, feat5

adata<-read.table("archaeadata.txt",header=T)
attach(adata)

prior = list(R = list(V = 1, nu = 0.002), G = list(G1 = list(V = 1, nu=0.002)))

m1<-
MCMCglmm(feat1~cluster,random=~animal,data=adata,pedigree=atree,prior=prior,verbose=FALSE,nitt=2
00000,thin=150,burnin=30000)

diag(autocorr(m1$VCV)[2, , ])
# this should be <0.1

summary(m1$Sol)
summary(m1$VC)
HPDinterval(m1$Sol)
# HPDinterval(m1$Sol,0.99)

# If zero is included in the interval
# then the clusters are not different, given the phylogeny

(m1$VCV/rowSums(m1$VCV))

m2<-
MCMCglmm(feat2~cluster,random=~animal,data=adata,pedigree=atree,prior=prior,verbose=FALSE,nitt=2
00000,thin=150,burnin=30000)
diag(autocorr(m2$VCV)[2, , ])
HPDinterval(m2$Sol)
(m2$VCV/rowSums(m2$VCV))
```

```
m3<-
MCMCglmm(feat3~cluster,random=~animal,data=adata,pedigree=atree,prior=prior,verbose=FALSE,nitt=2
00000,thin=150,burnin=30000)
diag(autocorr(m3$VCV)[2, , ])
HPDinterval(m3$Sol)
(m3$VCV/rowSums(m3$VCV))

m4<-
MCMCglmm(feat4~cluster,random=~animal,data=adata,pedigree=atree,prior=prior,verbose=FALSE,nitt=2
00000,thin=150,burnin=30000)
diag(autocorr(m4$VCV)[2, , ])
HPDinterval(m4$Sol)
(m4$VCV/rowSums(m4$VCV))

m5<-
MCMCglmm(feat5~cluster,random=~animal,data=adata,pedigree=atree,prior=prior,verbose=FALSE,nitt=2
00000,thin=150,burnin=30000)
diag(autocorr(m5$VCV)[2, , ])
HPDinterval(m5$Sol)
(m5$VCV/rowSums(m5$VCV))


########
########
```
